# Weak-Supervision Expectation–Maximization Framework for Identifying Decisional Vulnerability in Older Emergency Department Patients

**DOI:** 10.3390/healthcare14152394

**Published:** 2026-08-04

**Authors:** Devin Sandlin, Steve Arze, Jacob Lane, Ayan Bhakta, Jennifer A. Walker, Anirudh Rayanki, Jenna R. Williamson, Nathan Hoot, Hao Wang

**Affiliations:** 1 Department of Emergency Medicine, John Peter Smith Health Network, 1500 S. Main St., Fort Worth, TX 76104, USA; dsandlin@ies.healthcare (D.S.); jacobaustinlane@yahoo.com (J.L.); nhoot@ies.healthcare (N.H.); 2 Integrative Emergency Service, 4835 Lyndon B Johnson Fwy, Suite 900, Dallas, TX 75244, USA; sarze@ies.healthcare; 3 Data Science & Information Science, University of Illinois Urbana-Champaign, 601 East John St., Champaign, IL 61820, USA; ayanbhakta2006@gmail.com; 4 Department of Emergency Medicine, Baylor Scott and White All Saints Medical Center, 1400 8th Ave, Fort Worth, TX 76104, USA; jennifer.walker@bswhealth.org; 5 Naresh K. Vashisht College of Medicine, Texas A&M University College of Medicine, 3500 Gaston Avenue, Dallas, TX 75246, USA; anirudhrayanki@tamu.edu; 6 Anne Burnett Marion School of Medicine, Texas Christian University, 1100 W. Rosedale St., Fort Worth, TX 76104, USA; jenna.williamson@tcu.edu

**Keywords:** capacity, emergency department, geriatric, weak supervision

## Abstract

**Background and Objectives**: Decision-making capacity is essential for informed consent, yet its assessment in emergency departments (EDs) is often subjective and inconsistently documented. Older adults are particularly vulnerable to impaired capacity during acute illness. We aimed to develop a scalable, electronic health record (EHR)-based approach to support early identification of older ED patients at risk of decisional vulnerability using the Medical Information Mart for Intensive Care (MIMIC)-IV database. **Methods**: We conducted a retrospective cohort study of 51,195 ED patients aged ≥65 years. Clinicians manually reviewed 2000 patients using a conservative consensus protocol to establish a consensus-derived proxy for decisional vulnerability. Such an approach yielded a definitive reference subset (capacity vs. no capacity) and an “uncertain” category when consensus was not achieved. We developed a weak-supervision expectation–maximization (EM) label model that combined multiple noisy labeling functions derived from triage vital signs, acuity measures, arrival mode, and large language model (LLM)-classified chief complaints to estimate the probabilistic risk of impaired capacity. Model discrimination and calibration were assessed on an independent holdout subset of definitive reference labels using receiver operating characteristic area under the curve (ROC-AUC), precision–recall area under the curve (PR-AUC), calibration plots, and Brier score. To support clinically conservative use, operating thresholds were a priori constrained to limit automated flagging to ≤15% of patients, with the remaining ones deferred for clinician review. **Results**: On the definitive holdout set, the weak-supervision model achieved an ROC-AUC of approximately 0.855 and a PR-AUC of approximately 0.837. Calibration assessment demonstrated residual miscalibration in the generative posterior, which improved after a lightweight discriminative refinement step (logistic regression trained on EM-derived probabilistic labels), reducing the Brier score to approximately 0.224 on holdout evaluation. Under the prespecified operational constraint (≤15% auto-flagged), the model functioned as a conservative, selective alerting strategy, achieving high specificity and positive predictive value while identifying only a minority of patients with decisional vulnerability. **Conclusions**: This study demonstrates a methodological proof of concept for using weak supervision to model a retrospectively defined proxy for decisional vulnerability from routinely collected ED EHR data. The framework is intended to support conservative, triage-oriented prioritization. Further prospective validation, external testing, and workflow governance are needed before clinical implementation.

## 1. Introduction

Informed consent is a cornerstone of ethical and legal medical practice, ensuring that individuals understand their options, appreciate the implications, and voluntarily authorize their healthcare decisions [1,2]. The ability to provide informed consent depends on a person’s decision-making capacity, commonly referred to as mental capacity, which is defined as a person’s ability to maintain cognitive and psychological functions necessary to comprehend relevant information, appreciate the consequences, reason about treatment options, and communicate a choice [3,4]. When patients lose decision-making capacity, whether due to acute illness, delirium, dementia, psychiatric conditions, or medication effects, their ability to participate meaningfully in medical decisions becomes compromised [5,6]. Loss of capacity can have serious consequences, including delayed treatment, inappropriate care, ethical breaches, and the need for surrogate decision-making, which may not always reflect the patient’s true preferences [7].

The prevalence of decisional incapacity is substantial yet highly variable across populations. Reported estimates range from approximately 2.8% among healthy elderly people to 68% in patients with learning disabilities [3]. Older adults, who often present with complex comorbidities, cognitive impairment, or acute confusion, are particularly vulnerable to losing capacity during acute episodes of care [8]. In the high-pressure, time-sensitive Emergency Department (ED) environment where rapid decisions are often necessary, accurately screening patients who lack capacity is both critical and challenging. 

Current instruments designed to assess decisional capacity, such as the MacArthur Competence Assessment Tool for Treatment (MacCAT-T), the Aid to Capacity Evaluation (ACE), and the Hopkins Competency Assessment Test (HCAT), provide structured, validated frameworks for assessing capacity [3,9,10]. However, these tools are time-consuming, require specialized training, and are impractical in fast-paced emergency settings, where clinicians must make rapid judgments amidst competing priorities. Consequently, capacity assessments in the ED are often informal, subjective, and inconsistently documented [11,12]. Furthermore, most existing research on decisional capacity focuses on psychiatric, neurological, or inpatient populations, with limited evidence addressing capacity determination among older adults in emergency care. 

To date, very few standardized, scalable, and efficient tools exist to assist clinicians in systematically screening for decision-making incapacity in the ED. The CURVES mnemonic (Choose and Communicate, Understand, Reason, Value, Emergency, Surrogate) was developed to facilitate rapid assessment of medical decision-making capacity in acute settings [13]. While it is intended to be practical and easy to recall, there is limited direct evidence on its diagnostic accuracy or reliability compared to formal, validated capacity assessment instruments [13,14].

The lack of such tools carries profound implications. Underestimating incapacity can expose patients to interventions they cannot meaningfully consent to, violating the ethical principles of nonmaleficence and informed consent [3,5]. Conversely, overestimating incapacity may inappropriately limit patient participation in decision-making and risk undermining patient autonomy [6,15]. Given the growing aging population and the increasing complexity of care provided in EDs, there is a pressing need for rapid, objective, and data-driven approaches to help clinicians screen patients who may benefit from a more formal evaluation of decision-making capacity.

Despite longstanding ethical emphasis on informed consent, decision-making capacity assessment in the ED remains largely reactive rather than proactive. In routine practice, formal capacity evaluations are typically triggered only after overt concern arises, such as refusal of recommended care or evidence of severe cognitive impairment. This approach risks delayed recognition of decisional vulnerability, particularly in older adults with subtle, fluctuating, or context-dependent impairments. As a result, clinicians often face ethical uncertainty under time pressure, with limited support for early identification of patients who may benefit from more careful capacity assessment. This gap represents a critical unmet need in geriatric emergency care and motivates exploration of screening-oriented, data-driven approaches that can flag potential risk without replacing clinician judgment.

Recent advances in artificial intelligence (AI) and machine learning (ML) have enabled the development of predictive models that can extract clinical insights from large-scale electronic health record (EHR) datasets [16,17]. AI-based models have demonstrated success in predicting outcomes, such as delirium, sepsis, and mortality, often utilizing publicly available databases, including the Medical Information Mart for Intensive Care (MIMIC) [18,19,20]. However, to our knowledge, no prior studies have used AI/ML techniques to model decisional vulnerability or impaired capacity among geriatric patients in the emergency setting. The lack of such models may reflect both the conceptual challenges of operationalizing decisional capacity from EHR data and the limited availability of labeled datasets specifically addressing capacity assessments.

To address the challenge of limited labeled data, recent developments in weakly supervised learning frameworks have provided new opportunities for clinical prediction modeling [21,22]. In particular, expectation–maximization (EM) pipelines enable iterative refinement of probabilistic labels derived from noisy, indirect, or partially observed indicators, such as documentation patterns, cognitive assessments, and surrogate decision-making, which are commonly available in EHR data [21,22]. By combining clinician-derived heuristics or surrogate markers with unsupervised parameter estimation, weak-supervision models can approximate latent class probabilities in settings where direct annotations are scarce or costly to obtain [23]. This approach is particularly well-suited for modeling decisional vulnerability, where explicit documentation of capacity assessments is rare and often embedded within unstructured clinical notes [24]. Implementing an EM-based weak-supervision pipeline enables the model to leverage structured EHR features (e.g., demographics, comorbidities, vital signs, and triage characteristics) alongside indirect indicators of decisional vulnerability, thereby providing a scalable framework for exploratory modeling in emergency care.

Therefore, the objective of this study is to develop and evaluate a predictive framework to screen older adults (≥65 years) presenting to the ED who may be at increased risk of decisional vulnerability (a proxy for impaired decision-making capacity derived from retrospective EHR chart review), using retrospective data from the MIMIC-IV database. By leveraging routinely collected EHR data and integrating a weak-supervision EM pipeline, this study seeks to assess the methodological proof of concept and performance characteristics of an AI/ML framework for screening-oriented identification of decisional vulnerability. This approach is intended to inform future development of scalable, data-driven clinical decision support tools that prioritize conservative, uncertainty-aware triage support rather than autonomous capacity adjudication.

The primary methodological contributions of this study are threefold. First, we develop a weak-supervision EM framework to model a documentation-based proxy for decisional vulnerability in a setting where definitive capacity labels are scarce. Second, we incorporate an LLM-derived chief complaint labeling function (LF) to systematically leverage unstructured triage text within the weak-supervision framework. Third, we evaluate a conservative, clinician-supervised clinical decision support tool designed to identify patients who may benefit from further assessment while preserving clinician oversight and patient autonomy.

## 2. Methods

### 2.1. Study Design and Data Source

This study is a retrospective cohort analysis utilizing the Medical Information Mart for Intensive Care (MIMIC-IV, version 2.2) database. MIMIC-IV is a publicly available, de-identified dataset containing detailed health records of over 200,000 ED and intensive care unit (ICU) encounters from the Beth Israel Deaconess Medical Center (Boston, MA, USA) between 2008 and 2019 [25]. As the data are fully de-identified and publicly accessible, this study was exempt from institutional review board (IRB) oversight and was considered not human subjects research with informed consent waived (IRB#2235057-1).

### 2.2. Participants

The initial MIMIC-IV database includes 425,087 ED encounters. For this study, we first restricted the cohort to unique ED patients (n = 205,504, first encounter) and then further limited the sample to patients aged ≥65 years (n = 51,344). Patients were excluded if essential demographic information (e.g., age or sex) or outcome-related variables were missing (n = 149). The final analytic cohort consisted of 51,195 patients.

### 2.3. Outcome Measurement

The primary outcome of interest was decision-making capacity status during the ED encounter. Because no standardized, prospectively assessed measure of decision-making capacity is available in the MIMIC-IV dataset, we constructed a consensus-derived reference set through retrospective chart review.

A total of 2000 patient encounters were independently reviewed by three clinicians using a structured, conservative adjudication protocol. Reviewers classified each case into one of three categories: (1) lacking capacity, (2) retaining capacity, or (3) uncertain capacity. Cases were labeled as “uncertain” when documentation was insufficient to support a definitive classification or when unanimous agreement among reviewers was not achieved. Only cases with full consensus (capacity vs. no capacity) were considered definitive reference labels and were used for model evaluation and threshold selection; uncertain cases were excluded from discrimination and calibration analyses.

Adjudication was based on a comprehensive review of available ED documentation, including clinical presentation, vital signs, chief complaints, and diagnostic information. Indicators suggestive of impaired capacity included, for example, evidence of altered mental status, delirium, confusion, psychosis, or severe physiologic instability. Conversely, cases with a stable clinical presentation, no cognitive or psychiatric indicators, and no relevant history suggesting impaired decision-making were classified as retaining capacity. These criteria were intentionally conservative to prioritize label precision, which resulted in a relatively low prevalence of definitively labeled incapacity and a substantial proportion of uncertain cases.

Importantly, these reference labels do not represent a true ground truth of bedside decision-making capacity. Rather, they constitute a retrospectively defined proxy for decisional vulnerability, derived from documentation-based clinical indicators. As such, the modeled outcome reflects patterns observed in EHR documentation, serving as an initial screening signal to identify patients who may warrant further assessment of decision-making capacity.

Because several of the clinical features considered during manual chart review (e.g., vital signs, triage acuity, and chief complaint characteristics) overlap with variables used in the weak-supervision labeling functions, there is an inherent risk of incorporation bias. Consequently, the model may partially learn and reproduce the proxy signals used to construct the reference labels. This limitation should be considered when interpreting model performance.

Accordingly, the outcome in this study should be interpreted as an approximation of documentation-based decisional vulnerability, intended for screening-oriented, hypothesis-generating purposes. Such predictions are not final determinations or predictions for clinical decision-making.

### 2.4. Variables

The analytic dataset contained one record per encounter and included: (1) demographics: age, sex, and race/ethnicity (non-Hispanic White, non-Hispanic Black, Hispanic/Latino, and other); (2) clinical information: triage vital signs (temperature, heart rate, respiratory rate, oxygen saturation, systolic and diastolic blood pressure), Emergency Severity Index (ESI), mode of arrival (walk-in, ambulance/helicopter, or other), medication count at presentation, chief complaint, and ED diagnoses (ICD-10). Regarding medications, only antipsychotic and sedative agents with the potential to affect mental status were included in the analysis. Patients were flagged as potentially lacking capacity if their ICD-10 codes included terms related to altered mental status, confusion, delirium, dementia, disorientation, hallucinations, delusions, or Alzheimer’s disease. However, ICD-10 codes alone did not determine whether a patient’s decision-making capacity was lost. A comprehensive review of the patient was conducted by three independent reviewers, as addressed above. Although ICD-10 diagnostic categories were considered during manual chart review to support consensus labeling, they were not used as inputs in the weak-supervision model.

### 2.5. Classification of Chief Complaint Using a Large Language Model

Because the dataset included unstructured free-text entries, a large language model (LLM) was used to support structured categorization within the weak supervision framework. Specifically, the LLM was used to turn an unstructured free-text chief complaint into a structured categorical variable. No protected health information was entered into the LLM. The study used the fully de-identified MIMIC-IV database. Chief complaints contained no direct patient identifiers, and all analyses complied with MIMIC-IV data-use requirements. 

A subset of 2000 patient encounters was manually annotated by two independent reviewers (JL and AR), who categorized chief complaints into three groups reflecting potential decisional vulnerability signals—lacking capacity, retaining capacity, and uncertain capacity—based on full chart review. Inter-rater agreement was assessed using Cohen’s kappa statistic, and discrepancies were resolved by a third reviewer (HW). This manually reviewed subset was used to iteratively refine the LLM prompt and to assess agreement between LLM-generated outputs and human annotations.

The LLM (ChatGPT-5.0, OpenAI; accessed via the ChatGPT interface between June and December 2025) was used to classify chief complaint text into predefined categories corresponding to the three outcome classes. ChatGPT was selected because it provided high-quality natural-language understanding via an easily accessible interface, demonstrated strong agreement with manual annotations during prompt refinement, and required no task-specific model training [26]. Prompt development was conducted iteratively through multiple rounds of refinement using the manually annotated subset. The final prompt is provided in Appendix A. No additional system-level prompts beyond the default interface settings were applied.

Because the ChatGPT interface did not allow explicit control of parameters such as temperature, seed, or sampling configuration, these settings were not standardized. In repeated testing with the finalized prompt, outputs were consistent across runs; therefore, a single-pass classification approach was used for the full dataset. Agreement between LLM-generated classifications and the manually annotated subset was assessed using Cohen’s kappa statistic.

It is important to note that prompt refinement and evaluation were conducted on the same annotated subset, which may introduce optimism bias in agreement estimates. In addition, given limited control over model configuration and the absence of independent external validation, the LLM component should be interpreted as a pragmatic labeling function within the weak supervision framework.

### 2.6. Weak-Supervision Expectation–Maximization (EM) Pipeline

Data Pre-processing: Continuous features were normalized, categorical variables harmonized, and physiologic values clipped to plausible clinical ranges to reduce outlier influence: temperature: 86–120 °F, heart rate: 20–300 bpm, respiratory rate: 4–60/min, oxygen saturation: 50–100%, systolic BP: 30–300 mmHg, diastolic BP: 20–200 mmHg, age: 0–120 years, medication count: ≥0.

Weak Supervision and Labeling Functions: Because manual chart-level adjudication is resource-intensive, we employed weak supervision to identify the probability of consensus-defined decisional vulnerability. Multiple labeling functions (LF) were constructed from structured EHR features, including vital sign abnormalities, triage acuity measures, mode of arrival, and LLM-based classification of chief complaints. LFs were constructed from structured triage variables and LLM-derived chief complaint classifications. Each LF outputs a vote of +1 (suggesting no capacity), −1 (suggesting retained capacity), or 0 (abstain) based on predefined clinical heuristics (Supplementary Table S1). The LLM component served as an LF within the framework and did not independently determine final predictions. Under such conditions, the weak-supervision framework was designed to model this proxy. To evaluate the contribution of individual LFs and the robustness of the weak-supervision framework, we performed prespecified sensitivity analyses, including leave-one-labeling-function-out analyses and the exclusion of selected labeling-function domain models within individual LF groups. Performance was compared using ROC-AUC, PR-AUC, and Brier score (Supplementary Table S2).

Generative Label Model (Expectation–Maximization): We estimated the posterior probability of incapacity, p = Pr(no capacity), using a generative label model trained via the expectation–maximization (EM) algorithm. The EM model jointly estimated labeling-function sensitivities and specificities, the latent class prior, and patient-level posterior probabilities of impaired capacity. To evaluate dependence among labeling functions, we computed LF firing correlation matrices and signed agreement matrices. Pairwise signed agreement correlations were modest (maximum |r| < 0.30), indicating limited directional dependence among labeling functions (Supplementary Figure S1). The EM procedure was applied to the full cohort using only labeling-function outputs; definitive reference-standard labels were not used for parameter estimation. To enhance numerical stability, regularization strategies were incorporated, including parameter clipping and prior constraints.

Discriminative Refinement: We observed that the generative posterior probabilities exhibited discretization due to limited diversity in the LF patterns. To improve probability resolution and calibration, we implemented a lightweight discriminative refinement step. A logistic regression model was trained using EM-derived probabilistic labels as targets and structured EHR features as predictors. Reference-standard labels were not used for training this model. This refinement produced smoother posterior probability distributions and improved calibration performance, as assessed by the Brier score.

Data Partitioning and Threshold Selection: Definitive reference-standard labeled patients were stratified into a tuning subset (≈60%) and an independent holdout subset (≈40%). Threshold selection was performed exclusively on the tuning subset. Final discrimination (ROC-AUC, PR-AUC), calibration (Brier score), and threshold-based performance metrics were evaluated solely on the holdout subset. No holdout reference-standard labels were used for model parameter estimation or threshold optimization. This evaluation represented an internal validation using a withheld subset of the manually reviewed patients and did not constitute external validation.

Performance Evaluation: Model discrimination was assessed using the area under the receiver operating characteristic curve (ROC-AUC) and the area under the precision–recall curve (PR-AUC). Calibration was assessed using reliability plots and the Brier score. Bootstrap resampling (1000 iterations) was used to estimate 95% confidence intervals (CI) for discrimination metrics. At the selected operational threshold, additional classification metrics, including the confusion matrix, sensitivity, specificity, positive predictive value (PPV), negative predictive value (NPV), F1 score, accuracy, and three-class chief complaint classification performance, were calculated (Supplementary Table S3).

Operational Threshold Constraint: Because decisional capacity assessment carries ethical implications, operational thresholds were predefined to constrain automated flagging to ≤15% of encounters. Flagged patients were intended to prompt clinician reassessment rather than constitute automated adjudication. To facilitate interpretation of the operational deployment strategy, baseline characteristics of flagged and non-flagged patients were summarized (Supplementary Table S4). Because predictive values depend on disease prevalence, prevalence-adjusted PPV and NPV were additionally calculated across a range of assumed prevalences using the observed sensitivity and specificity at the selected threshold (Supplementary Table S5).

### 2.7. Statistical Analysis

Patients were categorized into three groups: Lacking Capacity, Retaining Capacity, and Uncertain Capacity. Baseline characteristics were summarized descriptively. Comparisons across the three groups were conducted using one-way analysis of variance (ANOVA) for continuous variables, reported as means with standard deviations, and the Chi-square test for categorical variables, reported as counts and proportions. A two-sided *p* value < 0.05 was considered statistically significant. Descriptive statistical analysis was conducted in STATA 14.2 (StataCorp, College Station, TX, USA), whereas the weak-supervision EM pipeline and performance metrics (including ROC-AUC and PR-AUC) were implemented in Python 3.8.

## 3. Results

### 3.1. Study Cohort Characteristics

The final cohort included 51,195 ED patients aged ≥65 years. A subset of 2000 patients underwent manual adjudication of decisional capacity. Of these, 708 patients received definitive consensus labels (capacity or no capacity), while the remainder were classified as uncertain due to insufficient documentation. Baseline demographic and clinical characteristics of the manually reviewed subset are summarized in Table 1. Across the three capacity groups, there were no statistically significant differences in sex, race/ethnicity, triage temperature, heart rate, or diastolic blood pressure. However, patients lacking capacity were older, were more frequently transported by ambulance (72.8%), and were triaged with higher acuity (ESI-1: 20.3%; ESI-2: 60.6%). These findings suggest that patients with consensus-defined decisional vulnerability tend to present with greater clinical instability and complexity at arrival.

### 3.2. Large Language Model Validation

Among the 2000 manually reviewed chief complaints, inter-rater reliability between the two independent reviewers was high (κ = 0.784, 95% CI 0.753–0.810, *p* < 0.001), indicating moderate-to-strong consistency in manual classification. The final ChatGPT-5.0 model also achieved stronger consistency (κ = 0.793, 95% CI 0.764–0.818, *p* < 0.001) when benchmarked against the reference-standard subset. This final prompt configuration was subsequently applied to classify chief complaints for the entire study cohort. 

### 3.3. Performance of the Weak-Supervision EM Model

**Figure 1:**
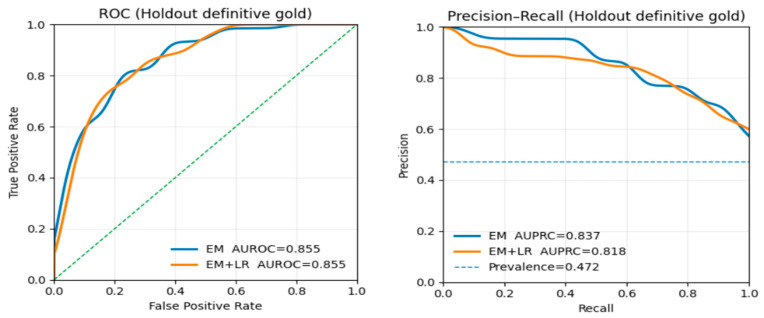
Model Discrimination on the Independent Holdout Set. Receiver operating characteristic (ROC) and precision–recall (PR) curves for the generative expectation–maximization (EM) label model evaluated on the independent holdout subset of definitively labeled encounters (n = 284). The EM model achieved an AUROC of 0.855 (95% CI 0.811–0.896) and an AUPRC of 0.837 (95% CI 0.776–0.894). Confidence intervals were estimated via bootstrap resampling (1000 iterations).

The expectation–maximization (EM) label model was trained using labeling-function outputs across the full cohort. On the independent holdout subset of definitively labeled patients (n = 284), the EM model achieved ROC-AUC of 0.855 (95% CI 0.811–0.896) and PR-AUC of 0.837 (95% CI 0.776–0.894, Figure 1). On internal validation using the independent holdout subset of definitively labeled patients, the model demonstrated moderate discrimination for identifying documentation patterns associated with decisional vulnerability. In addition, to evaluate the contribution of individual labeling functions, we performed a series of sensitivity analyses (Supplementary Table S2). Removal of the LLM-derived chief complaint labeling function reduced model discrimination (ROC-AUC 0.855 to 0.788), supporting its substantial contribution to overall performance. Removing both ESI and arrival-mode labeling functions further reduced performance (ROC-AUC 0.744). Interestingly, removing the vital-sign labeling function from the full model slightly improved discrimination and calibration, suggesting that this heuristic may have introduced additional noise rather than complementary information within the weak-supervision framework.

### 3.4. Calibration and Discriminative Refinement

**Figure 2:**
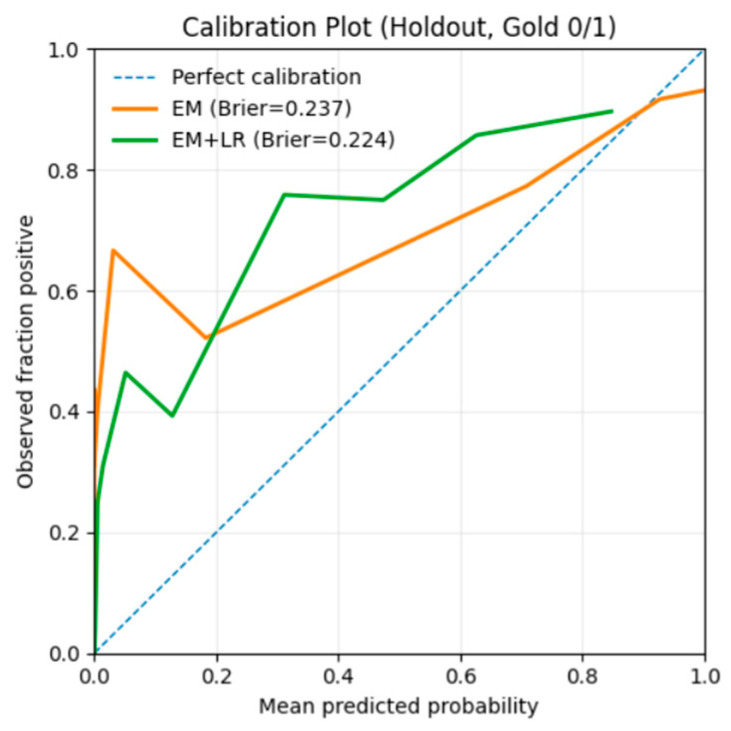
Calibration of Generative and Refined Models on the Independent Holdout Set. Calibration curves comparing the generative expectation–maximization (EM) model and the refined EM + LR model on definitively labeled holdout encounters (n = 284). The dashed diagonal represents perfect calibration. The EM posterior demonstrates residual miscalibration (Brier = 0.237), while logistic refinement improves calibration and probability resolution (Brier = 0.224), without materially altering discrimination.

**Figure 3:**
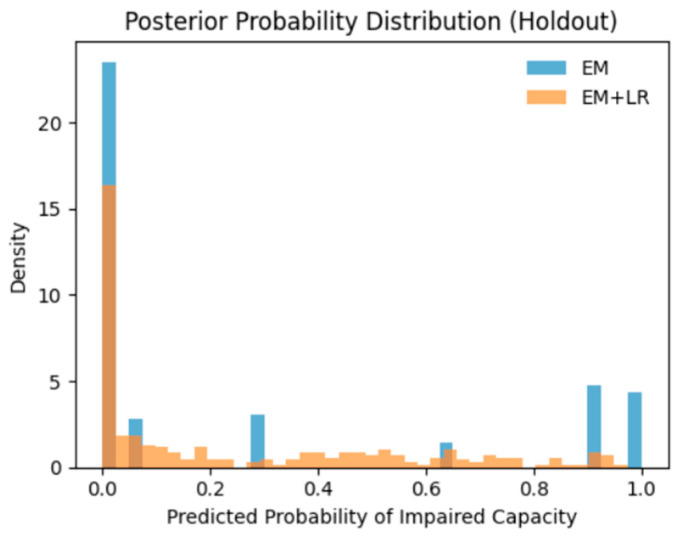
Posterior Probability Distributions. Posterior probability distributions on the independent holdout subset after discriminative refinement, enabling more stable operational threshold selection.

Calibration assessment on the holdout subset revealed residual miscalibration in the generative posterior, with a Brier score of approximately 0.237. To improve probability resolution and calibration, a logistic regression refinement model was trained using EM-derived probabilistic labels. On the independent holdout subset, the refined model achieved ROC-AUC of 0.855 (95% CI 0.808–0.897), PR-AUC of 0.818 (95% CI 0.747–0.885), and Brier score of 0.224 (Table 2). While discrimination remained similar, calibration improved following refinement (Figure 2). Posterior probability distributions after refinement exhibited substantially greater resolution, enabling stable operational threshold selection (Figure 3).

### 3.5. Operational Thresholding and Triage-Oriented Strategy

Threshold selection was performed using the tuning subset of definitive reference-standard labels, and the operational threshold corresponded to a probability cutoff of 0.635. On the independent holdout set, this threshold yielded 37 true positives, 7 false positives, 143 true negatives, and 97 false negatives, corresponding to a sensitivity of 0.276, specificity of 0.953, PPV of 0.8.14, NPV of 0.596, F1 score of 0.416, and overall accuracy of 0.615 (Supplementary Table S3). Applying the selected threshold to the entire analytic cohort identified 7680 of 51,195 patients (15.0%) as potentially having decisional vulnerability. Compared with non-flagged patients, flagged patients were more frequently transported by ambulance and more commonly had chief complaints categorized by the LLM as potentially lacking capacity, consistent with the intended conservative screening strategy (Supplementary Table S4). A total of 7680 patients were flagged. Flagged patients were intended to prompt clinician reassessment. This operating point reflects a conservative triage-support design that prioritizes minimizing false positives and protecting patient autonomy.

### 3.6. Decision Curve and Labeling Function Analysis

**Figure 4:**
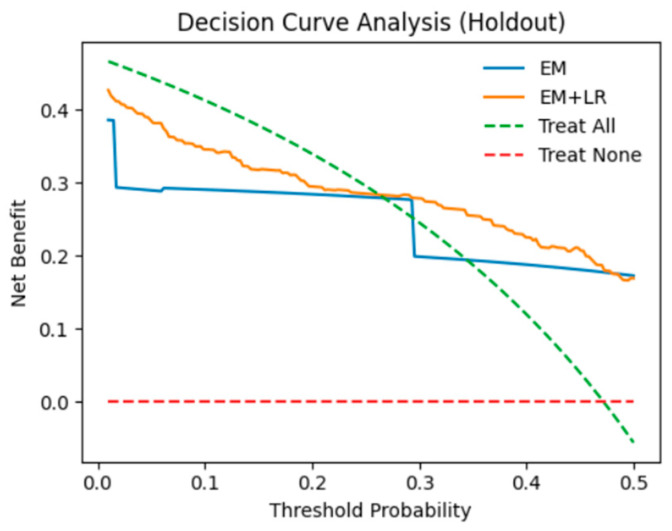
Decision Curve Analysis. Decision curve analysis comparing the EM and refined (EM + LR) models against treat-all and treat-none strategies on the independent holdout subset. Net benefit is shown across a range of clinically relevant probability thresholds. Both models demonstrated positive net benefit over default strategies within operationally meaningful thresholds.

Decision curve analysis demonstrated a positive net benefit across clinically relevant probability thresholds compared with the treat-all and treat-none strategies (Figure 4). LF correlation analyses showed moderate dependence among selected triage and physiologic indicators, but no single LF independently determined final predictions.

## 4. Discussion

In this study, we developed a weakly supervised machine learning framework to model a proxy signal of decision-making capacity using routinely collected EHR data in the ED. Using a consensus-derived reference set from retrospective chart review, the model demonstrated reasonable discrimination and calibration for identifying patients with documentation patterns suggestive of decisional vulnerability. Importantly, however, this work should be interpreted as a methodological proof of concept instead of a definitive predictive model of clinical decision-making capacity. A central finding of this study is that structured EHR data can be used to approximate signals of decisional vulnerability, enabling the development of a screening-oriented tool that flags a subset of patients who may warrant further clinical assessment. Under the prespecified operating threshold, the model achieved high specificity and positive predictive value, while maintaining low sensitivity. This performance profile indicates that the model functions as a narrow, selective alerting mechanism, prioritizing reduction in false-positive alerts over broad case detection. In practice, the framework is intended to operate after routine triage. Patients whose predicted probability exceeds the predefined threshold would be flagged for clinician reassessment of decision-making capacity. Thus, the model serves as a decision-support tool that prioritizes formal evaluation while preserving clinician oversight. Additionally, in the context of decision-making capacity where inappropriate labeling may carry ethical and clinical consequences, this conservative strategy may be appropriate for exploratory applications.

Recent AI studies have primarily focused on predicting sepsis, mortality, delirium, and intensive care unit outcomes using supervised learning [27,28]. In contrast, our study addresses a fundamentally different problem, modeling documentation-derived decisional vulnerability, using weak supervision in a setting where gold-standard labels are scarce. Thus, our contribution lies in demonstrating the feasibility of weak supervision instead of proposing a replacement for supervised models.

At the same time, several methodological considerations substantially limit the interpretation of these findings. Most importantly, the outcome used in this study is not a direct measure of bedside decision-making capacity. Instead, it represents a retrospectively defined, documentation-based proxy, derived from consensus review of EHR data using indirect clinical indicators. As such, the model does not learn or predict true underlying decisional capacity but rather approximates patterns in clinical documentation associated with perceived vulnerability.

Relatedly, retrospective EHR-based studies are inherently susceptible to several sources of bias, including selection bias, documentation bias, and incorporation bias. Among these, incorporation bias is particularly relevant because some of the clinical features considered during manual chart review, such as vital signs, triage acuity, and chief complaint characteristics, overlap with variables used in the labeling functions within the weak supervision pipeline. Consequently, the model may partially recapitulate the same proxy signals used to generate the reference labels. This limitation is intrinsic to retrospective EHR-based studies of unobservable clinical states and underscores the need for cautious interpretation.

The additional ablation analyses further illustrate that model performance was not uniformly dependent on any single labeling function. Although the LLM-derived chief complaint labeling function contributed substantially to discrimination, performance declined but remained above chance after its removal. Conversely, exclusion of the vital-sign labeling function slightly improved model performance, suggesting that not all heuristic labeling functions contributed equally. These findings emphasize that the weak-supervision framework integrated multiple imperfect signals rather than relying exclusively on a single documentation feature, while also highlighting opportunities for future refinement of labeling-function design. Additionally, because LLM-generated classifications may be imperfect, the LLM served only as one LF among multiple complementary LFs. Final predictions, therefore, reflected integration across multiple sources and did not depend on a single LLM output. Furthermore, agreement with manual review was evaluated using Cohen’s κ, and prompt refinements were made before application to the full cohort.

The predictive performance of the weak-supervision model, while improved relative to the initial analysis, must be interpreted cautiously. The model demonstrated moderate discrimination and acceptable precision–recall characteristics [29,30]. Calibration assessment revealed residual miscalibration in the generative EM posterior (Brier 0.237), which improved after a lightweight discriminative refinement step (Brier 0.224). This refinement did not materially alter discrimination but enhanced probability resolution, enabling more stable operational threshold selection [31]. These findings underscore that while weak supervision can recover informative signals from limited labels, careful calibration assessment is essential, particularly when modeling ethically sensitive constructs.

When applied to the full cohort under a prespecified operational constraint limiting automated flagging to ≤15% of patients, the model prioritized conservative triage support rather than universal screening. Importantly, flagged patients were intended to prompt clinician reassessment rather than constitute automated capacity adjudication. This design mitigates ethical concerns related to false positives by preserving clinician oversight and ensuring that algorithmic outputs remain advisory [32]. The divergence between the constrained prior in the EM model and operational flagging rates reflects thresholding decisions and should not be interpreted as a population-level estimate of incapacity.

A central innovation of this study is the application of a weakly supervised EM pipeline for modeling decisional vulnerability. Unlike traditional supervised learning, which requires large, labeled datasets, weak supervision leverages multiple noisy but complementary labeling functions to infer probabilistic labels [33]. This approach has been used in other clinical prediction tasks but has rarely been applied to ethical or cognitive constructions such as decisional capacity [33,34]. By enabling learning from incomplete and uncertain signals, including triage data, physiologic parameters, and structured interpretations of chief complaints, our framework demonstrates a scalable strategy for exploratory modeling of complex behavioral outcomes from existing clinical data.

This study has several strengths. First, it utilized the large, high-quality MIMIC-IV dataset, enabling systematic evaluation in a real-world ED cohort. Second, it implemented a weak-supervision EM framework specifically designed for limited-label settings. Third, the model relied on routinely collected variables available across most ED settings, supporting potential generalizability.

However, several limitations and implementation considerations warrant discussion. The analysis was conducted using a single-center dataset (MIMIC-IV), and the manually reviewed subset was derived from the same source population. Although a holdout set was used for evaluation, this represents internal validation only and does not establish external validity. Additionally, the relatively small number of definitively labeled cases and the substantial proportion of “uncertain” classifications reflect the inherent difficulty of assessing decision-making capacity from retrospective data, further limiting the strength of conclusions. In addition, the MIMIC-IV database includes encounters collected between 2008 and 2019. Although this database remains an extensively used resource for methodological development and benchmarking, changes in emergency care practices, documentation patterns, and patient populations over time may limit the direct applicability of the findings to contemporary clinical settings. Future studies should evaluate the proposed framework using more recent datasets.

Despite these limitations, this work highlights a potentially useful direction for future research. Instead of attempting to directly automate capacity determination, which is unlikely to be feasible or appropriate, data-driven approaches may support early identification of patients at risk of decisional vulnerability, prompting closer clinical evaluation. Future studies should focus on prospective validation using standardized bedside assessments, exploration of alternative labeling strategies, and integration of richer clinical and contextual data. Importantly, any such tools must be developed and evaluated within ethical frameworks that prioritize patient autonomy, minimize harm, and ensure appropriate clinician oversight.

## 5. Conclusions

This study presents a weakly supervised machine learning framework to model a documentation-based proxy for decisional vulnerability using routine ED data. The model demonstrates reasonable discrimination and a high-specificity performance profile, functioning as a selective alerting mechanism to identify a subset of patients who may warrant closer clinical assessment. However, the outcome represents a retrospectively derived proxy instead of true bedside decision-making capacity, and the potential for incorporation bias and lack of external validation limit interpretation and generalizability. Accordingly, these findings should be considered a proof of concept and hypothesis-generating. Future work should focus on prospective multicenter validation, external validation using independent datasets, and integration into clinician-supervised workflows before consideration of clinical implementation.

## Figures and Tables

**Table 1:** General Characteristics of the Manually Reviewed Patients.

	Having Capacity	Lacking Capacity	Uncertain Capacity
Number (%)	373 (18.7)	335 (16.8)	1292 (64.6)
Age—mean (SD)	75.1 (7.6)	78.7 (8.3)	76.3 (7.8)
Sex—n (%)			
Male	147 (39.4)	139 (41.5)	584 (45.2)
Female	226 (60.6)	196 (58.5)	708 (54.8)
Race/ethnicity—n (%)			
Non-Hispanic White	278 (74.5)	236 (70.5)	951 (73.6)
Non-Hispanic Black	39 (10.5)	45 (13.4)	147 (11.4)
Hispanic/Latino	12 (3.2)	9 (2.7)	42 (3.3)
Others	44 (11.8)	45 (13.4)	152 (11.8)
Temperature—mean (SD)	97.8 (3.3)	98.1 (1.1)	98.0 (2.0)
Heart rate—mean (SD)	80.3 (15.3)	83.0 (16.8)	80.9 (17.3)
Respiratory rate—mean (SD)	17.5 (2.0)	17.4 (2.3)	17.8 (2.6)
Pulse oximetry—mean (SD)	98.0 (2.0)	97.0 (5.5)	97.7 (2.2)
Systolic BP—mean (SD)	143.3 (23.6)	138.2 (25.7)	141.5 (25.9)
Diastolic BP—mean (SD)	75.9 (15.2)	73.9 (17.0)	82.3 (26.5)
Mode of arrival—n (%)			
Ambulance	128 (34.3)	244 (72.8)	709 (54.9)
Helicopter	2 (0.54)	1 (0.3)	8 (0.6)
Walk-In	242 (64.9)	61 (18.2)	512 (39.6)
Others	0 (0)	3 (0.9)	2 (0.2)
Unknown	1 (0.3)	26 (7.8)	61 (4.7)
Medication counts—mean (SD)	0.9 (1.2)	1.0 (1.5)	0.8 (1.3)
ESI—n (%)			
ESI-1	4 (1.1)	68 (20.3)	118 (9.1)
ESI-2	89 (23.9)	203 (60.6)	593 (45.9)
ESI-3	255 (68.4)	45 (13.4)	498 (38.5)
ESI-4	25 (6.7)	2 (0.6)	26 (2.0)
Unknown	0 (0)	17 (5.1)	57 (4.4)
Chief-complaint—n (%)			
Potentially retaining capacity	372 (99.7)	2 (0.6)	231 (17.9)
Potentially lacking capacity	0 (0)	333 (99.4)	83 (6.4)
Uncertain	1 (0.3)	0 (0)	978 (75.7)
ED Diagnoses ICD-10—n (%)			
Potentially retaining capacity	324 (86.9)	0 (0)	121 (9.4)
Potentially lacking capacity	2 (0.5)	297 (88.7)	68 (5.3)
Uncertain	47 (12.6)	38 (11.3)	1103 (85.4)

Abbreviations: n, number; SD, standard deviation; BP, blood pressure; ESI, emergency severity index; ICD-10, international classification of diseases, tenth revision.

**Table 2:** Model Performance on Independent Holdout Set (Definitive Labels Only).

Model	ROC-AUC (95% CI)	PR-AUC (95% CI)	Brier Score
EM	0.855 (0.811–0.896)	0.837 (0.776–0.894)	0.237
EM + LR	0.855 (0.808–0.897)	0.818 (0.747–0.885)	0.224

## Data Availability

The data analyzed in this study are publicly available from the MIMIC-IV (Medical Information Mart for Intensive Care IV) database hosted on PhysioNet (https://physionet.org). Access to data is subject to completion of required credentialing and data use agreements.

## Supplementary Materials

The following supporting information can be downloaded at: https://www.mdpi.com/article/10.3390/healthcare14152394/s1, Figure S1. Correlation structure among labeling functions (LFs). (A) Pearson correlation of LF firing indicators (non-abstain events). (B) Pearson correlation of signed LF outputs (no capacity = +1, capacity = −1, abstain = 0). These matrices summarize dependence across LFs and provide transparency into the conditional-independence assumptions used in the generative label model. Table S1. Definition and Operationalization of Labeling Functions in the Weak-Supervision Model. Table S2. Label-Function Ablation Analyses. Table S3. Classification Metrics Reports. Table S4. Baseline Characteristics of Flagged versus Non-flagged Patients. Table S5. Prevalence Adjusted PPV and NPV at the Selected Operational Threshold.

## Appendix A. LLM Prompt for Chief Complaint Classification

Task: Using only the information contained in the uploaded file, classify each chief complaint in column “C” into one of three categories and add the classification to a new column “D” named “capacity_cc”.

Output Coding: 1 (“incapable”): The chief complaint explicitly suggests possible impairment of cognition, consciousness, orientation, psychosis, or decisional function. 0 (“capable”): The chief complaint describes a primarily localized, somatic, or non-cognitive condition with no explicit indication of impaired mental status. 99 (“uncertain”): The chief complaint does not clearly indicate either category, or contains insufficient information to determine capacity relevance.

Definitions: Class 1 (“incapable”) should be assigned only when the chief complaint explicitly includes terms or phrases strongly associated with impaired cognition, awareness, or decision-making. Examples include (but are not limited to): altered mental status (AMS), confusion, disorientation, psychosis, dementia, lethargy, coma, encephalopathy, delirium, hallucination, delusion, Alzheimer’s disease, unresponsive, decreased level of consciousness. Assignment to class 1 should require an explicit textual indication of cognitive or consciousness impairment.

Class 0 (“capable”) should be assigned when the chief complaint clearly reflects a localized physical complaint without mention of cognitive or mental-status concerns. Examples include (but are not limited to): ear pain, nosebleed, toothache, cough, medication refill, musculoskeletal pain (e.g., wrist pain, knee pain), localized swelling, and eye irritation. Assignment to class 0 should require the absence of cognitive or mental-status terms.

Class 99 (“uncertain”) should be assigned when the complaint is ambiguous (e.g., “weakness,” “dizziness” without mental-status context). The complaint could be associated with either cognitive or non-cognitive causes. The complaint lacks sufficient information to determine decisional relevance.

Additional Instructions: Use only the text in column “C”. Do not infer beyond the explicit chief complaint wording. Do not assume diagnosis or clinical course. When in doubt, classify as “99” (uncertain). Do not use external knowledge beyond general clinical language understanding. Do not prioritize minimizing false positives or false negatives; follow the explicit criteria above. After classification, output the file with the additional column “capacity_cc”.
