# Supplementary material for: Weak-Supervision Expectation–Maximization Framework for Identifying Decisional Vulnerability in Older Emergency Department Patients
Source: Healthcare (Basel). 2026 Aug 4;14(15):2394. doi: 10.3390/healthcare14152394 (PMC13465668; doi:10.3390/healthcare14152394)
Supplement: Supplementary file 1 [file healthcare-14-02394-s001.zip › healthcare-4301024-supplementary.pdf]

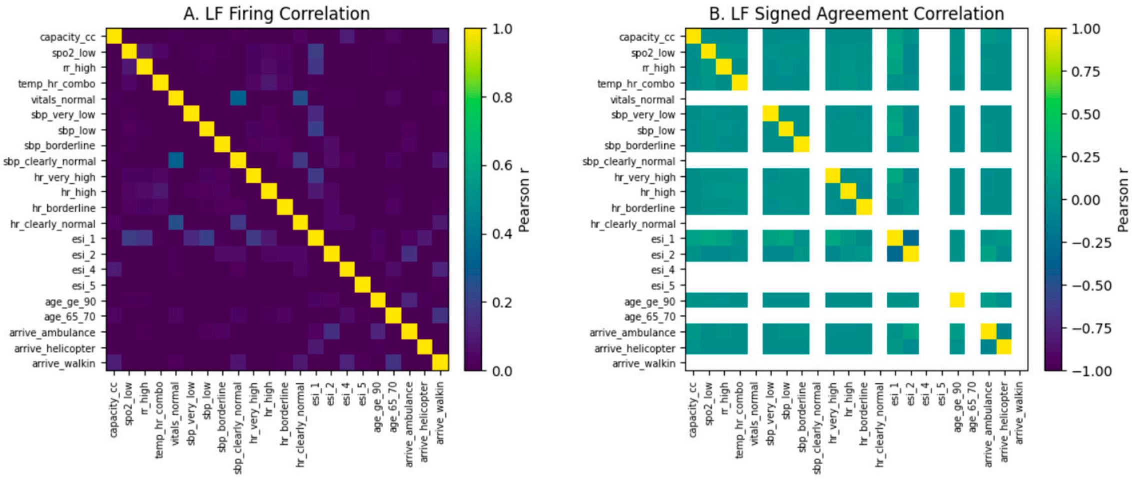

Figure S1. Correlation structure among labeling functions (LFs). (A) Pearson correlation of LF firing indicators (non-abstain events). (B) Pearson correlation of signed LF outputs (no capacity = +1, capacity = −1, abstain = 0). These matrices summarize dependence across LFs and provide transparency into the conditional-independence assumptions used in the generative label model.

Table S1. Definition and Operationalization of Labeling Functions in the Weak-Supervision Model

Labeling Function	Input variables	1 (no capacity)	-1 (capacity)	0 (abstain)	Clinical rationale
LF-vital signs	Temp, HR, RR, BP, POx	Any physiologic value outside predefined abnormal thresholds (e.g., Temp>102.5F° or <96F°; HR >120 or <45 bpm; SBP <90 or >160mmHg; DBP<60 or >100mmHg RR >30 or <6 per min; Pox<94%.	All vital signs within normal clinical ranges (Temp 98-100.3F°; HR 60-99; RR 8-19, SBP 90-139mmHg, DBP 60-89mmHg, Pox≥94%.	Temp (96-97.9F°, or 100.4-102.5F°; HR (100-120, or 45-59), RR (6-7 or 20-30), SBP (140-160mg), DBP (90-100mmHg).	Physiologic instability may be associated with acute illness, delirium, or impaired cognition
LF-ESI acuity	ESI 1-5	ESI 1-2	ESI 4-5	ESI-3 or unknown	Higher triage acuity reflects more severe illness, which may correlate with impaired cognition or decisional capacity

LF-arrival_mode	Mode of arrival	Ambulance or helicopter	Walk-in	Others or unknown	Emergency transport is often associated with acute or severe conditions that may affect mental status
LF-chief-complaint-LLM	LLM-classified chief complaints	Classified as no capacity	Classified as having capacity	Classified as uncertain	Chief complaints provide indirect information on cognitive status; LLM classification standardizes the interpretation of unstructured text
LF-medication	Sedative/antipsychotic medication count	≥3	0	1-2 or unknown	Certain medications may affect mental status and decision-making ability

Table S2. Label-Function Ablation Analyses

Model	ROC-AUC	PR-AUC	Brier Score
Full model	0.855	0.837	0.237
Model without LLM-chief complaint LF	0.788	0.753	0.266
Model without ESI and arrival modes LFs	0.744	0.746	0.434
Leave one domain out:			
Model without LLM-chief complaint	0.788	0.753	0.266
Model without vital signs	0.885	0.883	0.157
Model without ESI	0.809	0.807	0.315
Model without arrival mode	0.815	0.797	0.317

Table S3. Classification Metrics Reports

Binary holdout evaluations	Value	Three-class chief complaint classification metrics	Value
Operational threshold	0.635	Retain capacity (n)	6,057
Number of Holdout (n)	284	Precision	0.235
Holdout positive prevalence	0.472	Recall	0.372
True Positive (n)	37	F1-score	0.288
False Positive (n)	7	Decisional vulnerability (n)	3,190
True Negative (n)	143	Precision	0.382
False Negative (n)	97	Recall	0.398
Sensitivity/Recall (positive decisional vulnerability)	0.276	F1-score	0.390
Specificity	0.953	Uncertainty (n)	41,948
PPV	0.841	Precision	0.857
NPV	0.596	Recall	0.781
F1 score	0.416	F1-score	0.817
Accuracy	0.615	Overall accuracy	0.709

Table S4. Baseline Characteristics of Flagged versus Non-flagged Patients

Variable	Flagged vs. Non-flagged	Category	Number	Percentage
Sex	Non-flagged	Female	23,432	53.9
	Non-flagged	Male	20,083	46.2
	Flagged	Female	4,406	57.4
	Flagged	Male	3,274	42.6
Race/ethnicity	Non-flagged	NHW	32,085	73.7
	Non-flagged	NHB	4,975	11.4
	Non-flagged	Hispanic	1,399	3.2
	Non-flagged	Others	5,056	11.6
	Flagged	NHW	5,661	73.7
	Flagged	NHB	591	7.7
	Flagged	Hispanic	113	1.5
	Flagged	Others	1,315	17.1
Mode of arrival	Non-flagged	Ambulance	21,556	49.5
	Non-flagged	Helicopter	41	0.1
	Non-flagged	Other	74	0.2
	Non-flagged	Unknown	1,930	4.4
	Non-flagged	Walk-in	19,914	45.8

	Flagged	Ambulance	6,847	89.2
	Flagged	Helicopter	246	3.2
	Flagged	Other	4	0.1
	Flagged	Unknown	509	6.6
	Flagged	Walk-in	74	1.0
LLM-chief complaint	Non-flagged	Potentially retaining capacity	9,188	21.1
	Non-flagged	Potentially lacking capacity	2,309	5.3
	Non-flagged	Uncertainty	32,018	73.6
	Flagged	Potentially retaining capacity	425	5.5
	Flagged	Potentially lacking capacity	1,020	13.3
	Flagged	Uncertainty	6,235	81.2

Table S5. Prevalence Adjusted PPV and NPV at the Selected Operational Threshold

Assumed Prevalence Percentage	Sensitivity from the holdout threshold	Specificity from the holdout threshold	Prevalence-adjusted PPV	Prevalence-adjusted NPV
1%	0.276	0.953	0.056	0.992
2%	0.276	0.953	0.108	0.985
5%	0.276	0.953	0.237	0.962
10%	0.276	0.953	0.397	0.922
20%	0.276	0.953	0.597	0.840
30%	0.276	0.953	0.717	0.754
47%	0.276	0.953	0.841	0.596
